# The influence of temporal frequency and stimulus size on the relative contribution of luminance and L-/M-cone opponent mechanisms in heterochromatic flicker ERGs

**DOI:** 10.1007/s10633-021-09837-9

**Published:** 2021-04-22

**Authors:** Jan Kremers, Avinash J. Aher, Yassen Popov, Maziar Mirsalehi, Cord Huchzermeyer

**Affiliations:** Section for Retinal Physiology, University Hospital Erlangen, Schwabachanlage 6, 91054 Erlangen, Germany

**Keywords:** Heterochromatic modulation, Electroretinography, Luminance, L-/M-cone opponency, Vector addition, Harmonics, Temporal frequency, Stimulus size

## Abstract

**Purpose:**

To study the effect of stimulus size and temporal frequency on the relative contribution of luminance and L-/M-cone opponent signals in the ERG.

**Methods:**

In four healthy, color normal subjects, ERG responses to heterochromatic stimuli with sinusoidal, counter-phase modulation of red and green LEDs were measured. By inverse variation of red and green contrasts, we varied luminance contrast while keeping L-/M-cone opponent chromatic contrast constant. The first harmonic components in the full field ERGs are independent of stimulus contrast at 12 Hz, while responses to 36 Hz stimuli vary, reaching a minimum close to isoluminance. It was assumed that ERG responses reflect L-/M-cone opponency at 12 Hz and luminance at 36 Hz. In this study, we modeled the influence of temporal frequency on the relative contribution of these mechanisms at intermediate frequencies, measured the influence of stimulus size on model parameters, and analyzed the second harmonic component at 12 Hz.

**Results:**

The responses at all frequencies and stimulus sizes could be described by a linear vector addition of luminance and L-/M-cone opponent reflecting ERGs. The contribution of the luminance mechanism increased with increasing temporal frequency and with increasing stimulus size, whereas the gain of the L-/M-cone opponent mechanism was independent of stimulus size and was larger at lower temporal frequencies. Thus, the luminance mechanism dominated at lower temporal frequencies with large stimuli. At 12 Hz, the second harmonic component reflected the luminance mechanism.

**Conclusions:**

The ERGs to heterochromatic stimuli can be fully described in terms of linear combinations of responses in the (magnocellular) luminance and the (parvocellular) L-/M-opponent retino-geniculate pathways. The non-invasive study of these pathways in human subjects may have implications for basic research and for clinical research.

## Introduction

The electroretinogram (ERG) is an electrophysiological response of the retina to visual stimulation and is an important non-invasive clinical tool to study the functional integrity of the retina [1]. Although the ERG originates in the retina, it is difficult to associate flash ERG responses with the responses of single neurons and with psychophysical data [2] despite some resemblances between ERGs to monochromatic flashes and their detection thresholds [3]. In recent years, there is growing evidence that ERG responses to continuous sinusoidal stimulation containing both luminance and red-green chromatic modulation, can reflect the activity of the luminance (putatively magnocellular) and the L-/M-cone opponent (putatively parvocellular) retino-geniculate pathways. The ERGs, however, probably originate in activity of the bipolar cells [4]. We previously proposed that the midget bipolar cells have similar physiological properties as the midget retinal ganglion cells belonging to the L-/M-opponent sensitive parvocellular pathway. Additionally, we suggest that diffuse bipolar cells are luminance sensitive similar to the parasol retinal ganglion cells (belonging to the magnocellular pathway) [5, 6]. Which of the two pathways dominates in the ERG response depends on the stimulus size and on its temporal frequency. Luminance reflecting ERG responses to sine wave modulations are particularly large at temporal frequencies of 30 Hz and higher [5, 7, 8] and with full field stimuli [9, 10], whereas cone opponent responses dominate at temporal frequencies below 16 Hz and with spatially restricted stimulus sizes up to about 70° in diameter. The dominance of cone opponent reflecting ERGs with small stimulus sizes is caused by the absence of a strong dependency of their amplitudes on stimulus size and position. The amplitudes of luminance reflecting ERGs, however, decrease strongly with decreasing stimulus size and distance to the fovea [9–11].

At intermediate temporal frequencies, the ERGs may reflect a combination of luminance and cone opponent activities and it was shown that they can be well described by a linear vector addition of pure luminance and pure cone opponent responses. With full field stimuli, the relative contribution of the luminance responses increases with increasing temporal frequency, whereas the amplitude of the cone opponent response is negatively correlated with temporal frequency [8]. The influence of stimulus size on the interaction between two ERG pathways is not known and a precise description of the combined influence of temporal frequency and stimulus size is lacking. Since stimulus size influences the amplitudes of the two ERG pathways differently, it can be expected that it will have an effect on their interaction. It is the purpose of the present study to come to a detailed description of the interaction between the two pathways at different temporal frequencies and different stimulus sizes. More specifically, we wanted to study if the response interactions can be completely explained by a simple linear vector addition for all stimuli. Alternatively, the interactions between the responses of the two pathways may involve nonlinearities. Furthermore, we wanted to describe the amplitude and timing properties of the two pathways in conditions, where it can be expected that both substantially contribute to the total response.

The second harmonic components in the ERG responses to 12 Hz sine-wave stimuli display properties of the luminance system, because its amplitude is minimal at isoluminance [5, 12, 13]. However, its origin may also be a nonlinearity in the cone opponent ERG responses [5]. The ERG measurements at different stimulus sizes may be a way to distinguish between the two possibilities: If the second harmonic component originates in the luminance pathway, then it should depend strongly on stimulus size. If its origin is in the L-/M-cone opponent pathway, the amplitude can be expected to be similar at different stimulus sizes. In the present study, we therefore also describe the second harmonic amplitudes to 12 Hz sine-wave stimuli at different stimulus sizes and compare them with the luminance and cone opponent pathways in the fundamental components of the ERGs.

## Methods

### Subjects

Four healthy subjects (one female; three male authors; age between 26 and 61 years) participated in this study. The subjects received a full ophthalmological investigation. They had normal color vision as established with an anomaloscope. The subjects were informed and signed a written consent before ERG measurements. The study followed the tenets of the Declaration of Helsinki and was approved by the ethics committee of the Friedrich-Alexander University Erlangen-Nürnberg. The total recording time for each subject was about seven hours performed in several sessions lasting maximally 1.5 h.

### ERGs measurements

ERG recordings were performed on the right eyes of the subjects using the RETIport system (Roland Consult GmbH, Germany). The eyes were dilated with a drop of 0.5% tropicamide (Stulln Pharma GmbH, Germany) prior to recording. The left eyes were occluded using an eyepatch during the recordings. A fiber electrode, placed over the lower conjunctiva and attached to the inner and outer canthus, served as the active electrode. The skin on the forehead and the ipsilateral temple was cleaned with NuPrep® abrasive gel (D.O. Weaver & Co., Colorado, USA). Two gold cup electrodes, filled with electrode paste (DO weaver & Co.) served as ground (forehead) and reference (ipsilateral temple), respectively. The impedance of the active and reference electrodes was below 5 KΩ. The signals were amplified 100,000 times, band-pass filtered between 1 and 300 Hz, and sampled at 2048 Hz. The ERG responses from 80 sweeps (two trials of 40 sweeps), each lasting 1 s, were averaged. To avoid onset artifacts, the first 2 s of recording time after start of the stimulation were discarded.

### Visual stimuli

**Fig. 1:**
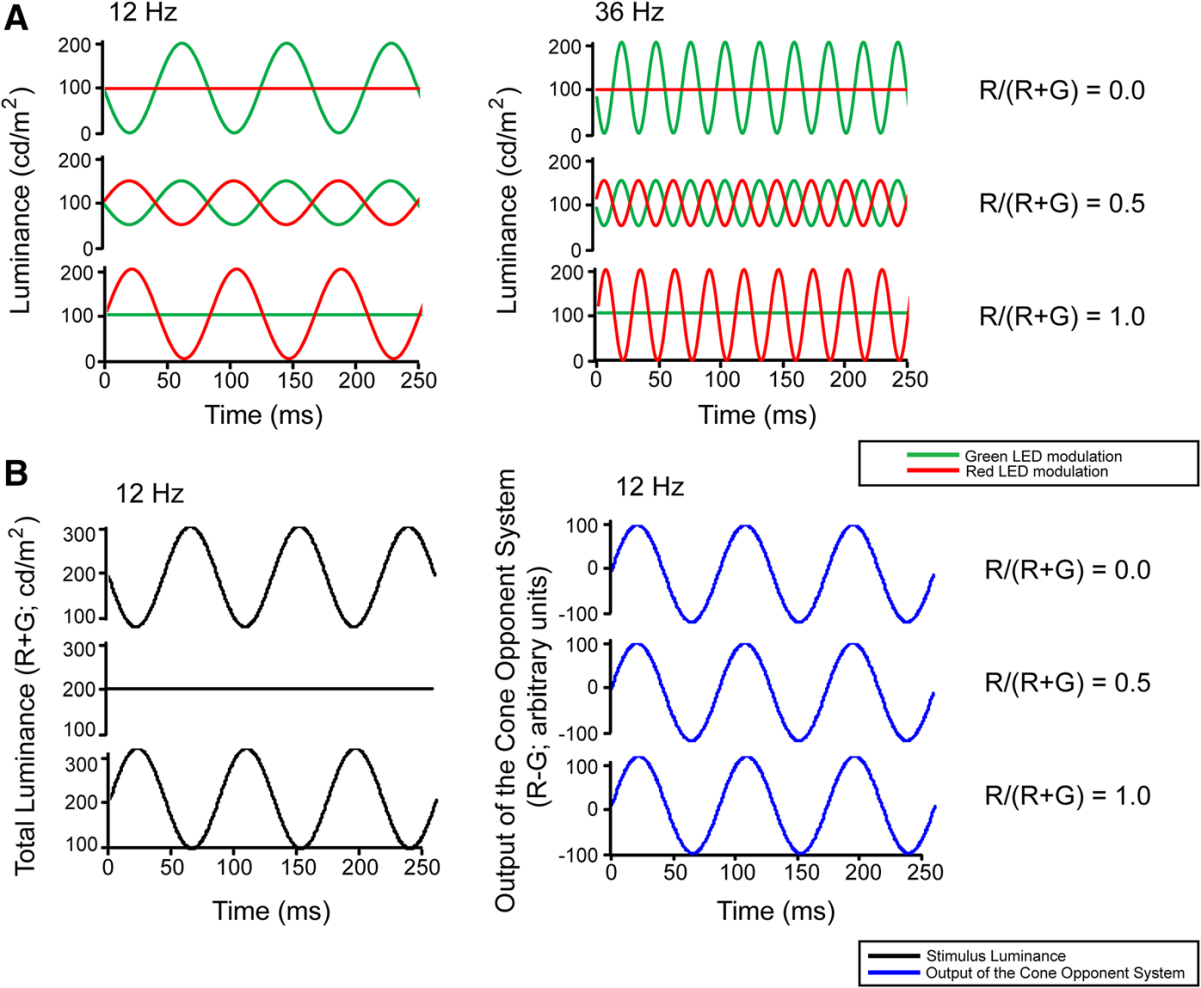
**a** Representation of the heterochromatic stimuli. Modulation in the red and green stimuli in a 250 ms period is displayed for 12 Hz (left) and 36 Hz (right) frequencies. Three conditions are shown with *F*_R_ values at 0, 0.5 and 1.0. **b** The resultant luminance (left) and chromatic (right) modulation for the 12 Hz stimuli. From Barboni et al. [12]; copyright: Association for Research in Vision and Ophthalmology

Heterochromatic stimulation was created by counter-phase sinusoidal modulation of red and green LED array outputs using the Q450SC Ganzfeld stimulator (Roland Consult GmbH, Germany). The stimuli are described in detail elsewhere [8, 12]. Briefly, the mean luminances of the red and green LEDs were 100 cd/m^2^ each. The Michelson contrast in the red and green stimuli (*R* and *G*, respectively) were varied without changing the total contrast (*R* + *G* = 100%). As a result, the fraction of red contrast (*F*_R_ = *R*/[*R* + *G*]) was varied between 0 (no red modulation; 100% contrast green modulation) and 1 (100% contrast red modulation; no green modulation). At *F*_R_ = 0.5, red and green contrasts were 50%. A representation of the stimuli and its consequences for luminance and chromatic modulation are shown in Fig. 1. Eight different values of F_R_ were used: 0, 0.2, 0.3, 0.4, 0.5, 0.6, 0.8 and 1.0. The measurements were performed at 13 different temporal frequencies between 12 and 36 Hz (in 2 Hz steps).

This series of measurements was repeated for three different stimulus sizes: full field (FF) and spatially restricted circular stimuli with 70° and 30° diameter. The spatially restricted stimulus fields were created by apertures in black cardboard positioned at 3 cm distance from the observer's eyes. A detailed description of the stimuli can be found elsewhere [9]. Although the edges of the stimuli were extremely blurred, we checked that the stimuli indeed only stimulated a part of the retina and that the mean retinal illuminance was not affected by the size of the aperture [11].

### Data analysis

One sec averages of the ERG responses were Fourier transformed using an FFT and the amplitudes and phases of the first harmonic (fundamental) components were extracted. All analyses were performed on these components. From the 12 Hz responses, the second harmonic components were also extracted for describing the dependency on *F*_R_ and stimulus size. The response phases were accepted for further analysis only when the signal-to-noise ratio (SNR) was larger than 2. The noise was defined as an average of amplitudes at F − 1 Hz and F + 1 Hz, where F Hz is a fundamental frequency. For example, the noise at 12 Hz was defined as the average of amplitudes at 11 Hz and 13 Hz.

## Results

**Fig. 2:**
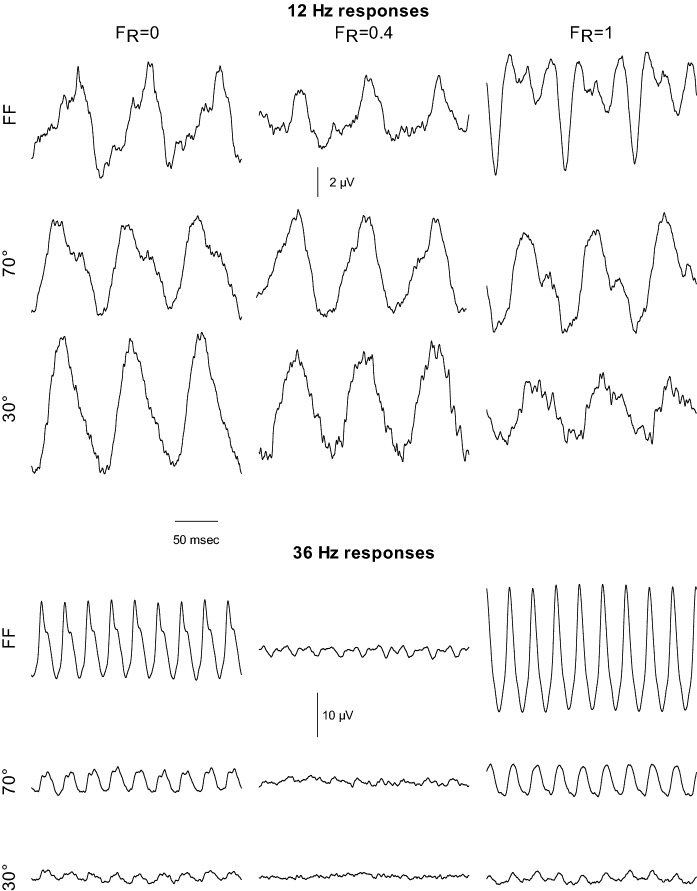
Averaged original ERG responses to red-green heterochromatic modulations with *F*_R_ = 0 (left column), 0.4 (middle) and 1 (right) in subject JK. The responses were averaged in 250 ms episodes. The responses to 12 Hz (upper three rows) and 36 Hz (lower three rows) stimuli are shown, each for full field (FF), 70° diameter and 30° diameter stimulus sizes. Please observe the different vertical scale bars for the 12 and 36 Hz responses

The original ERG responses measured in one representative subject in 250 ms episodes (obtained by additional averaging of the 1 sec recordings) at *F*_R_ values 0, 0.4 and 1 are displayed in Fig. 2 at 12 and 36 Hz and the different stimulus sizes. The following observations are of interest: First, at 36 Hz the responses at *F*_R_ = 0.4 are small for all stimulus sizes whereas large responses were found for *F*_R_ = 0 and 1. Close inspection shows that the responses at *F*_R_ = 0 and F_R_ = 1 are phase shifted relative to each other by about 180 deg for all stimulus sizes. Second, the 36 Hz responses are generally smaller with smaller stimuli. Third, at 12 Hz, the responses do not show a minimal response and the response timing is similar for all recordings. Neither F_R_ nor stimulus size has a large influence on the response amplitude although the response waveform does vary. Fourth, the 12 Hz responses are not all sinusoidal, particularly with large stimuli.

### First harmonic components

**Fig. 3:**
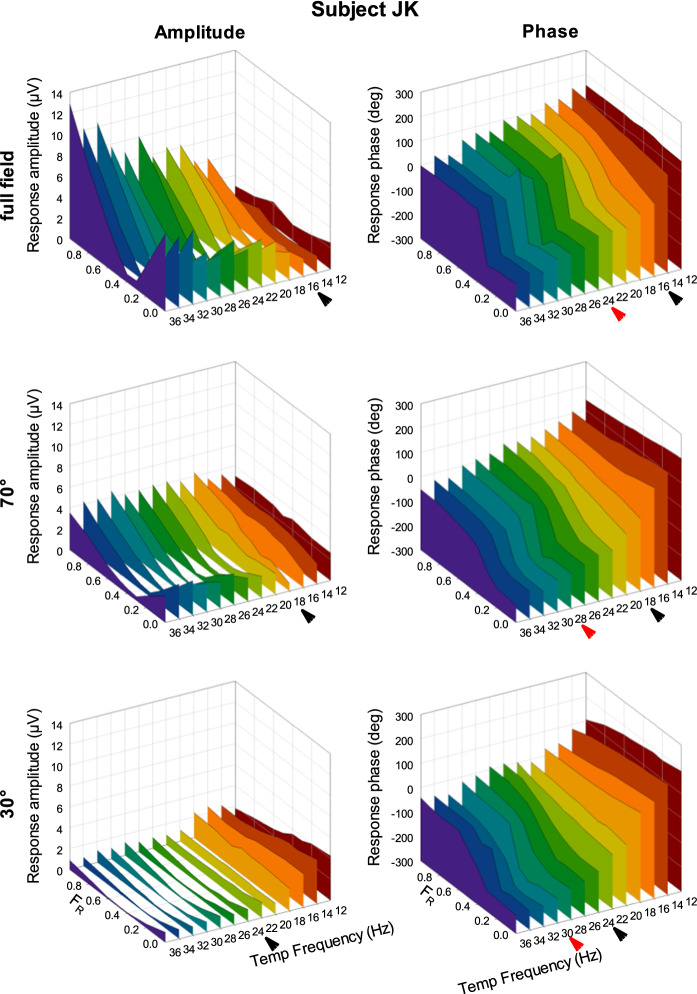
Response amplitude (left plots) and phase (right plots) measured in subject JK plotted versus *F*_R_ and temporal frequency and shown separately for full field (FF; upper row), 70° (middle row) and 30° diameter (lower row) stimuli. The arrowheads display the lower (black) and upper limits of transition ranges in the response properties

**Fig. 4:**
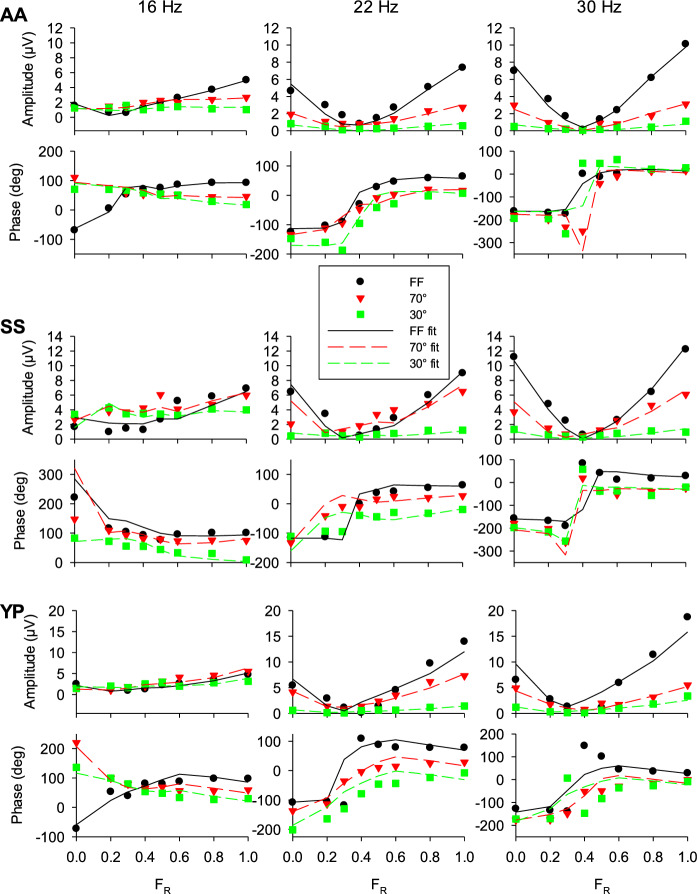
Response amplitudes and phases in three subjects (AA upper two rows; SS 3rd and 4th rows; YP lower two rows) at 16, 22 and 30 Hz. Each plot displays the data for the three stimulus sizes together with the fits of a vector addition of the FF 12 Hz and FF 36 Hz responses (FF: data black circles, fits black drawn lines; 70°: data red inverted triangles, fits red long dashed lines; 30°: data green squares, fits green short dashed lines)

The ERG responses were Fourier analyzed. In Fig. 3, the amplitudes (left plots) and phases (right plots) of the first harmonic (fundamental) components, measured in subject JK, are plotted as a function of F_R_ and temporal frequency. The results are plotted separately for full field (upper plots), 70° diameter stimuli (middle plots) and 30° diameter stimuli (lower plots). These data are representative for the responses measured in the other subjects (additional data are shown in Fig. 4). Furthermore, they are in agreement with results of previously performed recordings [8, 10, 12]. We are therefore confident that the effects, found in the four subjects who participated in the present study, are general features. The response amplitudes at 36 Hz depend strongly on *F*_R_ and are minimal at *F*_R_ ≈ 0.4. At the minimum, there is a sharp phase change of about 180°. The V-shaped amplitude and the sharp phase changes can be observed for all stimulus sizes. However, the response amplitudes strongly decreased with decreasing stimulus size. In contrast, the response amplitudes and phases at 12 Hz did not change as strongly with *F*_R_ and with stimulus size. These results are also in agreement with the results of previous measurements [10].

At intermediate temporal frequencies, the response amplitudes and phases display a gradual transition between those found at 12 and 36 Hz. The black arrowheads show the lowest frequency where the amplitudes displayed a partial V-shape *F*_R_ dependency and where a substantial phase change is observed. The red arrowheads mark the lowest frequency, where the phase change is abrupt. It can be seen that the range of transition in response characteristics shifts toward higher temporal frequencies when stimulus size decreases. This qualitative observation will be quantified for all observers below. Another observation is that the phases decrease with increasing temporal frequency for all values of *F*_R_ and for the three stimulus sizes. This observation was made for all four participants and will also be formalized below.

We previously modeled the responses to full field stimuli at intermediate temporal frequencies by a vector addition of the responses at 12 and 36 Hz [8] representing putative L-/M-opponent chromatic and luminance mechanisms, respectively. Here, we use the same model to describe the responses at different temporal frequencies and different stimulus sizes as a vector addition of the full field 12 Hz and 36 Hz responses. The details of the vector addition model and the fitting procedure are described previously [8, 14]. Briefly, the responses were presented as vectors in polar plots with the lengths of the vectors representing the response amplitudes and the angles with the positive abscissa representing the response phases. For each temporal frequency and stimulus size, the vectors at the different F_R_ values were fitted by the addition of the response vectors obtained with the full field stimuli at 12 Hz and at 36 Hz by minimizing the sum of squared distances (in vector space) between the measured response vectors and the vector additions of the FF 12 Hz and 36 Hz vectors. In the fits (using the “solver” routine in Excel), there were four free parameters: An amplitude gain and a phase shift in the 12 Hz and in the 36 Hz FF responses (assuming that the 12 Hz and 36 Hz input signal need to be multiplied by a factor -here called “gain”- and shifted in time, which we called “phase shift” before they are vector added to obtain a response description at another frequency). Figure 4 displays responses from the remaining three subjects at three different frequencies (16, 22 and 30 Hz) and with different stimulus sizes with the resulting fits. The responses of these subjects indicate that the above described features for subject JK were generally present. The fits are generally satisfactory although the responses to high frequency 30° stimuli were often small with F_R_ values between 0.2 and 0.5. As a result, their phases were not always reliable and did not strongly constrain the fits.

**Fig. 5:**
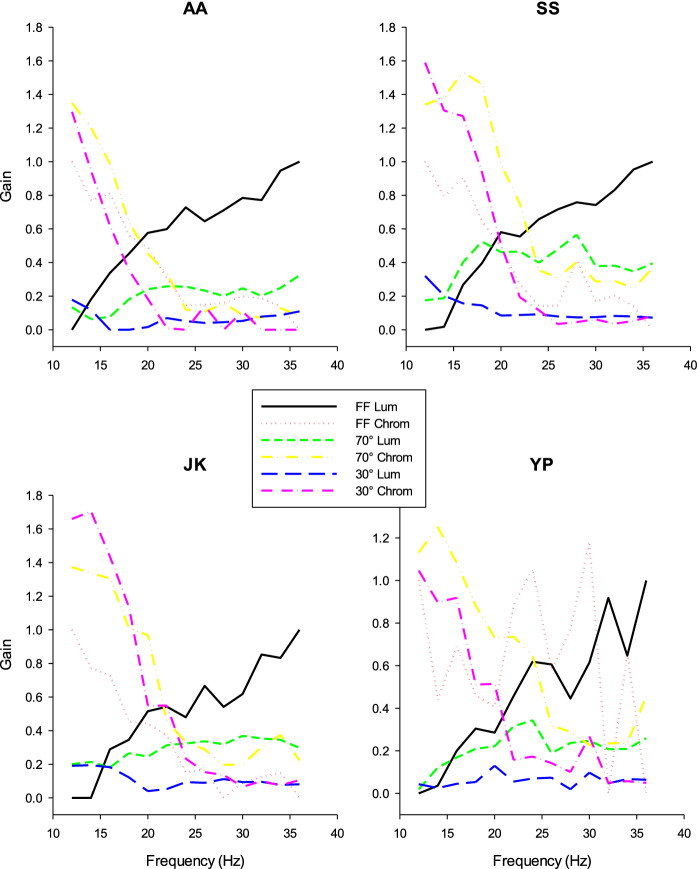
Estimated gains of the luminance (FF 36 Hz; “Lum”) and cone opponent chromatic (FF 12 Hz; “Chrom”) responses, obtained from the fits of the vector addition model to the measured ERG data, plotted as a function of temporal frequency. The results are plotted separately for the three stimulus sizes. The four panels show the results for the four subjects who participated in the present study

As mentioned above, the responses of the 36 Hz and the 12 Hz FF responses (putatively reflecting luminance and chromatic pathways, respectively) were multiplied by gains that were free parameters in the fits. The estimated gains, obtained from the fits of the vector addition model to the data, are plotted vs. temporal frequencies in Fig. 5 and shown separately for the different stimulus sizes and for the four subjects. For all the subjects, the gains of the FF 12 Hz (chromatic) ERG mechanism decreased and those of the FF 36 Hz (luminance) ERG mechanism increased with increasing temporal frequency. The stimulus size had relatively little influence on the gains of the FF 12 Hz mechanism. The gains of the FF 36 Hz decreased strongly with decreasing stimulus size. At low temporal frequencies, the gains of the FF 36 Hz mechanisms were slightly larger than those at intermediate temporal frequencies with 30° stimuli. We attribute this to intrusion of rod-driven responses through stray light [13]. These effects were found for all four subjects.

**Fig. 6:**
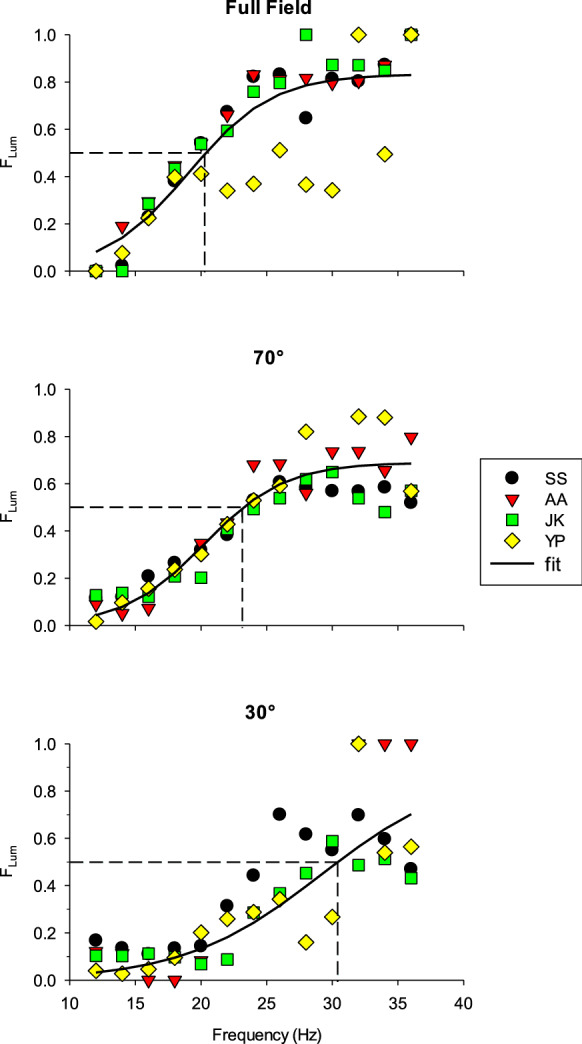
Fraction of the gain of the luminance pathway in the total gain [*F*_Lum_ = *G*_Lum_/(*G*_Lum_ + *G*_Chrom_)] as a function of temporal frequencies. The data from all subjects were fitted with a sigmoid function. The three plots represent the results obtained with the different stimulus sizes (Full field: upper plot; 70°: middle plot; 30°: lower plot). The dashed lines indicate the frequency where *F*_Lum_ equals 0.5

To pursue this issue in more detail, we calculated the fraction of the FF 36 Hz (luminance) gain (*G*_Lum_) in the total gains (i.e., the sum of the gains of the FF 12 Hz and the FF 36 Hz mechanisms): *F*_Lum_ = *G*_Lum_/(*G*_Lum_ + *G*_Chrom_). These were plotted as a function of temporal frequency (see Fig. 6). The fractions have similar characteristics for the different subjects and the accumulated data at each stimulus size were fitted with sigmoidal curves that are described as follows:

$$F_{{{\text{Lum}}}} = \frac{{{\text{Max}}\left( {F_{{{\text{Lum}}}} } \right)}}{{\left( {1 + \exp \left( {R \times \left( {{\text{Freq}}_{0.5 \times \max } - {\text{Freq}}} \right)} \right)} \right)}}$$

where Max(*F*_Lum_*)* is the maximal luminance fraction, *R* is a constant defining the frequency range where *F*_lum_ changes, *Freq* is the temporal frequency and Freq_0.5 × max_ is the frequency at which *F*_Lum_ equals 0.5 × max*(F*_Lum_*)*. In the fits, there were three free parameters (Max*(F*_Lum_*)*, *R* and Freq_0.5_).

The fits revealed that the frequency where *F*_Lum_ = 0.5 (i.e., where the luminance and chromatic driven signals have equal strengths [*G*_Lum_ = *G*_Chrom_], indicated by the dashed lines in Fig. 6) was 20.4 Hz at FF, 23.2 Hz with 70° and 30.4 Hz with 30° stimuli.

**Fig. 7:**
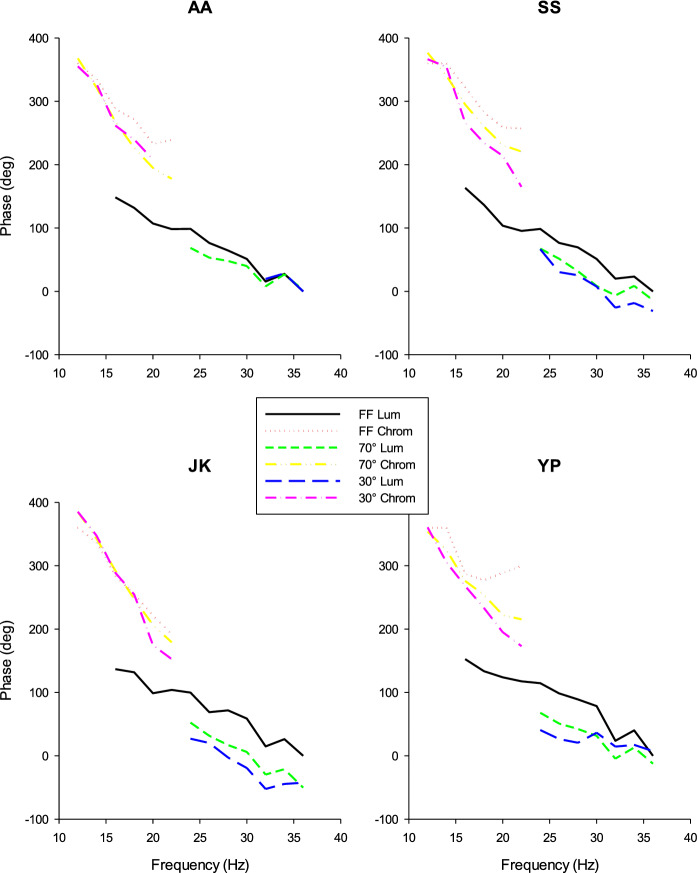
Response phases of the luminance (FF 36 Hz; “Lum”) and cone opponent chromatic (FF 12 Hz; “Chrom”) responses in the fits to the measured responses vs. temporal frequencies and plotted separately for the three stimulus sizes. The four panels show the results for the four subjects who participated in the present study

We also extracted the phase shifts of the two ERG pathways from the fits of the vector addition model to the ERG response data. However, with low gains, the phase shifts are unreliable. We therefore considered the phases if the gains were larger than 0.08. The phases of the chromatic ERG pathway at frequencies ≤ 22 Hz were taken into consideration. Phases of the luminance ERG pathway were used at frequencies ≥ 16 Hz for FF stimuli and at frequencies ≥ 24 Hz for spatially restricted stimuli. Figure 7 displays the resultant phases. Again, the phase vs. frequency were quite consistent in the different subjects: First, the absolute phases were very similar. Second, the phase plots of the chromatic ERG pathway were similar for all stimulus sizes, but they were shifted relative to each other in the luminance pathways. Third, for all conditions, the phases depended in an approximately linear manner on temporal frequency. We therefore performed linear regression through the data (results not shown). The slopes of the linear regressions were shallower for the luminance ERG pathway in comparison with those of the chromatic ERG pathway. Linear phase vs. frequency relationships can be fully explained by response latencies and from the slopes the apparent latencies were calculated [15, 16]. We found that the apparent latencies were on average 47 ms (n = 12 four subjects and three stimulus sizes each; between 21 and 68 ms) for the chromatic ERG pathway and 18 ms (*n* = 12; between 6 and 23 ms) for the luminance ERG pathway. The data indicate that the apparently latency in the chromatic ERG pathway increased (i.e., the slopes of the phase vs. frequency plots became steeper) with decreasing stimulus size (mean apparent latency FF: 35 ms, 70°:50 ms and 30°:58 ms). The apparent latency did not depend on stimulus size in the luminance ERGs because the slopes were similar for all subjects (mean apparent latency FF: 20 ms, 70°:18 ms and 30°:15 ms) although their absolute phases seemed to decrease by maximally about 80 deg when size was decreased from FF to 30°.

### Second harmonic components

**Fig. 8:**
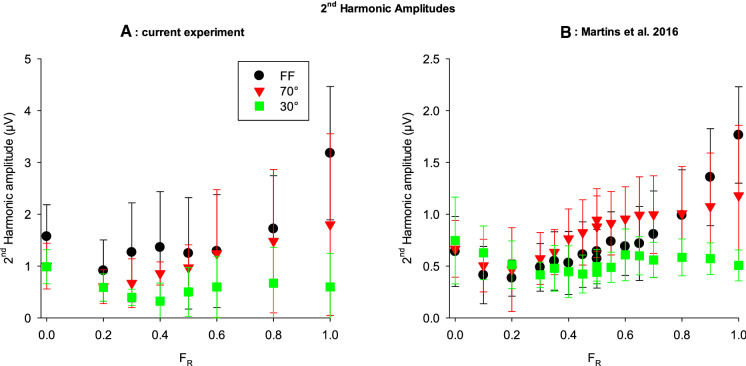
The amplitude of the second harmonic component in the 12 Hz responses as a function of F_R_ plotted separately for the three stimulus sizes. A (left): Current study; B (right): data obtained from the measurements performed by Martins et al. [10]

It was previously shown that the second harmonic component at 12 Hz shows a V-shaped amplitude dependency on F_R_ in the stimulus [12, 17], similar to the first harmonic components to 36 Hz responses. A dependency of the second harmonic components to 12 Hz stimuli on stimulus size was not studied yet. Figure 8a displays the amplitudes of the second harmonic components as a function of *F*_R_ plotted separately for the FF, 70° and 30° stimuli. In agreement with the previous data, we found a strong dependency on F_R_ with a minimum. Furthermore, the amplitude of the second harmonic component decreased with decreasing stimulus size. We performed similar recordings in a previous study with five subjects (two of whom—authors AA and JK participated also in the present study) to a larger number of *F*_R_ values and stimulus sizes [10] where we did only considered the first harmonic components. We now extracted the second harmonic components from the previous measurements at the same stimulus sizes as used in the present study. The data are shown in Fig. 8b and they confirm the results of the present study.

## Discussion

The purpose of the present study was to describe in detail the influence of temporal frequency and stimulus size on the interaction between ERGs reflecting L-/M-cone opponent and luminance activity probably originating in parvo- and magnocellular retino-geniculate pathways, respectively. Particularly, we were interested to study if a linear interaction between the two ERG mechanisms was sufficient to describe the ERG responses or whether additional mechanisms or nonlinear interactions should be taken into account. Finally, we wanted to study the influence of stimulus size on the second harmonic component at 12 Hz that was found to be substantially large with full field stimuli [16–18].

### First Harmonic components

The data show that all responses can be sufficiently and completely described by a linear vector addition of two previously described ERG mechanisms: one that reflects L-/M-cone opponency and one that reflects luminance activity. This is in agreement with previous preliminary findings [8]. The influence of the cone opponent mechanism decreases with increasing temporal frequency and is independent of stimulus size (within the range of sizes used in the present experiments; obviously the response amplitudes should decrease with very small stimuli, because in the extreme case where the stimulus becomes infinitely small or absent, a response should also be absent). The contribution of the luminance mechanism increases with increasing temporal frequency and increasing stimulus size. These findings confirm previous findings [9, 10, 15, 16] and extend these findings to frequency ranges and stimulus sizes in which both mechanisms contribute substantially to the total ERG response. The result is that the frequency range of transition from L-/M- cone opponent dominated to luminance dominated ERGs is shifted toward larger values when stimulus size is decreased.

Stimulus size and frequency also influence the response phase. The linear relationship between phase and temporal frequency indicates that the phases are mainly determined by an apparent delay that can be determined from the slopes of the linear regression through the phase vs. frequency data. The apparent delays were substantially larger for ERGs reflecting L-/M-opponency than for luminance ERGs and with full field stimuli they were comparable to those found previously [16].

The apparent delays of the cone opponent ERGs were consistently larger for smaller stimuli in all four subjects (as indicated by the steeper phase change as a function of frequency; see Fig. 7). This effect may be related with the previously reported implicit time increase of b-wave-like and PhNR-like components in the responses to L-cone-On and M-cone-Off stimuli with decreasing stimulus size [19]. The change in apparent delay with stimulus size suggests an eccentricity-dependent alteration in cone opponent processing. It is possibly related to the finding that the optimal temporal frequency to isoluminant red-green modulation in L-/M-opponent parvocellular ganglion cells increases with increasing retinal eccentricity [20]. However, both peripheral magno- and parvocellular ganglion cells have higher optimal frequencies and respond phase advanced relative to central cells to luminance modulation, indicating that their response delay is smaller in peripheral cells [21]. This could therefore explain the decreased apparent delay in ERGs with large stimuli where peripheral responses are included. On the other hand, the expectation would then be that the apparent delays of the (magnocellular reflecting) luminance ERGs would also differ. This was not the case. Instead, the absolute phase increased with decreasing stimulus size. Possibly, the frequency range in the current experiment is too small (24–36 Hz, whereas the frequencies in the single-cell recordings varied between 0.61 and 78 Hz) to reveal slope differences in the luminance ERG. Additional data are needed to draw firmer conclusions.

In standard flash or flicker ERGs, generally, luminance stimuli are used [1]. As a result, the ERGs reflect only luminance mechanisms. In addition, the use of flashes may substantially limit the informative value of the ERG. With flashes, energy is compressed in a short time and may bring the retina into a mode of operation that is not physiological [22]. The use of continuous stimuli may be more informative about retinal pathways in a physiological state.

Chromatic flashes on a desensitizing background of a different chromaticity are used to bias the response originating in a particular photoreceptor [23, 24] and/or a particular component such as the PhNR [25, 26]. However, different chromatic backgrounds bring the retina in different states of adaptation thereby prohibiting a direct comparison of the results in different conditions. Furthermore, the stimulus strengths (in terms of Michelson contrast or Weber fraction) at the level of the different photoreceptor types are not always easy to quantify for the different spectral compositions and a perfect isolation of photoreceptor responses is not possible. Finally, there are indications that the differences in PhNR amplitudes to different chromatic combinations may not be large if the stimuli are equated for the luminance contents [27]. By using continuous stimuli that contain red-green chromatic modulation in the stimulus (for instance the heterochromatic modulations as used in the present paper or L- and M-cone isolating stimuli employing a silent substitution paradigm), the ERGs can be recorded at different (cone or rod) contrasts without changing the state of adaptation and may provide information about underlying mechanisms. Furthermore, perfect isolation is at least theoretically possible. They therefore may open up additional opportunities for future investigations. They can provide information about color vision in human subjects [17, 28] and about physiological changes in the presence of a disease that affects the retina [13]. Finally, the underlying luminance and L-/M-cone opponent mechanisms have properties that resemble those of the magnocellular and parvocellular retino-geniculate pathways [5, 7, 8] providing an opportunity to study these pathways, that are relevant for visual perception, non-invasively in human subjects.

### Second Harmonic components

The dependency of 12 Hz second harmonic component amplitudes on F_R_ resembles that of the first harmonic amplitude in responses to high temporal frequency stimuli (cf. Figure 8 with Figs. 3 and 4; see also Aher et al. [17]) with a minimum close to isoluminance. This indicates that the second harmonic component originates in luminance activity and does not originate in a nonlinearity in the cone opponent responses [5]. This notion is now strengthened by our finding that, similar to the luminance reflecting ERG, the second harmonic component at 12 Hz becomes smaller when stimulus size decreases. It is therefore possible to efficiently study the properties of the opponent and luminance ERGs solely by measuring the responses to 12 Hz FF stimuli through extraction of the first and second harmonic components.

The question why the second harmonic component is related to luminance activity is a matter of discussion. We propose that luminance and chromatic ERG signals are superimposed at 12 Hz. If the stimulus contains luminance modulation then the two signals are relatively large and a second harmonic ERG is revealed. At isoluminance, only the chromatic ERG is revealed resulting in a small second harmonic component. Interestingly, a similar superposition of signals has been found for responses to high contrast luminance stimuli [18] that possibly causes a minimum in the first harmonic response and a maximum in the second harmonic component at 12 Hz [29–31].

## Conclusion

Our findings add to the knowledge of retinal processing and of the components underlying flicker ERGs. Furthermore, the demonstration of a linear summation of the L-/M-opponent and the luminance mechanisms in the flicker ERG and the clarification of the influence of stimulus size allows creating clinically useful protocols for objective determination of functional losses in these retino-geniculate systems. Using the second harmonic at 12 Hz might help to reduce measurement time.

## Acknowledgements

The authors like to thank Sarah Stolper for her support.

## Declarations

### Statement on the welfare of animals

No animals were used in the present study.
